# Editorial: Regulation of Inflammation, Its Resolution and Therapeutic Targeting

**DOI:** 10.3389/fimmu.2017.00415

**Published:** 2017-04-18

**Authors:** Mariagrazia Uguccioni, Mauro Martins Teixeira, Massimo Locati, Alberto Mantovani

**Affiliations:** ^1^Institute for Research in Biomedicine, Università della Svizzera Italiana, Bellinzona, Switzerland; ^2^Laboratorio de Imunofarmacologia, Departamento de Bioquimica e Imunologia, Instituto de Ciencias Biologicas, Universidade Federal de Minas Gerais, Belo Horizonte, Brazil; ^3^Humanitas Clinical and Research Center, Rozzano, Italy; ^4^Department of Medical Biotechnologies and Translational Medicine, University of Milan, Milan, Italy; ^5^Humanitas University, Rozzano, Italy

**Keywords:** regulation, inflammation, therapeutic targeting, cell migration, TLR

**Editorial on the Research Topic**

**Regulation of Inflammation, Its Resolution and Therapeutic Targeting**

Inflammation underlies the pathogenesis of diverse human diseases ranging from infection, immune-mediated disorders to cardiovascular pathology, neurodegeneration, and cancer. Progress in the field of immunity and inflammation has led to a change in paradigm concerning resolution. Resolution has emerged as an integrated actively orchestrated process with multiple players. These include metabolites of the arachidonic acid cascade, anti-inflammatory cytokines, decoy, and scavenger receptors. Inflammatory cells undergo genetic reprograming during resolution with for instance orientation of macrophages to a pro-resolving mode. Failure of resolution has emerged as a fundamental mechanism of disease. Smoldering non-resolving inflammation is, for instance, an essential constituent of the tumor microenvironment. Under self-limiting conditions, inflammation spontaneously resolves in an active process. Some cellular and molecular mechanisms involved in inflammation resolution have been uncovered in the recent past and include generation of specific cytokines, apoptosis of inflammatory leukocytes, lipid mediators, macrophage repolarization, and others are likely to be revealed in the next future, since loss-of-function mutations of an increasing number of genes results in the development of spontaneous inflammation in experimental animals. “Pushing for” resolution of inflammation by exploiting active naturally occurring pro-resolving processes may have significant advantages over the attempt to simply “push back” inflammation by passive blockade of pro-inflammatory mediators.

This Topic of Frontiers offers the reader views on key aspects of the regulation of resolution of inflammation. Its foundations lay in the European Union supported project TIMER (Targeting novel mechanisms of resolution in inflammation)^1^ and the effort of the International Union of Immunological Societies (IUIS)^2^ that have fostered the collaboration of several groups in Europe and Brazil, supporting the preclinical work on molecules and mechanisms involved in resolution of inflammation as a basis for the development of innovative therapeutic strategies in chronic inflammation and autoimmune disease. These studies have been laid the foundations in the clinical trials that have been initiated.

The topic is placed in the context set by a review from Sugimoto et al. that focuses on the events required for an effective transition from the pro-inflammatory phase to the onset and establishment of resolution, suggesting that the mediators promoting inflammation can simultaneously propel the program for an active resolution. A set of articles then focus on specific molecular players involved in this process. Molgora et al. summarize evidence indicating IL-1R8 as a key anti-inflammatory molecule, which needs further investigation in human pathology as its targeting holds promise of innovative therapies in several inflammatory conditions. Alves-Filho and Palsson-McDermott review expression and enzymatic activities of PKM2 that can be regulated at multiple levels, including transcription, posttranslational modifications, and allosteric regulation of conformational stability. PKM2 represents a novel potential target for the development of anti-inflammatory drugs, as recent studies have unraveled a notable involvement of PKM2 in controlling the transcriptional activity of HIF-1α and STAT3 pathways during inflammation (Alves-Filho and Palsson-McDermott). Still on STAT3, Muhl reviews the preclinical data suggesting that providing recombinant STAT3-activating cytokines directly targeting hepatocytes, especially IL-11 and IL-22, may evolve as additional novel pro-regenerative therapeutic option in hard-to-treat patients where standard therapy with *N*-acetylcysteine alone falls short. Notably, the benefit of focused short-term application of IL-11 or IL-22 in acute disorders, such as APAP-induced ALI, should likely outweigh the inherent danger of these cytokines to promote in the long run tumor growth, which has been detected for IL-22 and hepatocellular carcinoma patients (Muhl). Proudfoot and Uguccioni discuss how synergy between chemokines or DAMP molecules, together with the low-affinity interaction with GAGs can tune the response of leukocytes to chemokines, controlling leukocyte extravasation into damaged or inflamed tissues. Dampening inflammation targeting the chemokine system can be achieved either targeting chemokines or their receptors. Blood-sucking parasites inhibit the recruitment of immune cells by producing a class of chemokine-binding proteins known as Evasins, whose advantages and disadvantages for potential development for therapeutic use is here discussed by Bonvin et al. On the receptor side, allosteric antagonists of chemokine receptors, discussed by Allegretti et al., might provide both functional selectivity and probe/concentration dependence. Vertebrates have adopted a number of mechanisms for removing chemokines from inflamed sites to help precipitate resolution. Over the past 15 years, it has become apparent that essential players in this process are the members of the atypical chemokine receptor (ACKR) family. Broadly speaking, this family is expressed on stromal cell types and scavenges chemokines to either limit their spatial availability or to remove them from *in vivo* sites. Here, Bonecchi and Graham provide a brief review of these ACKRs and discuss their involvement in the resolution of inflammatory responses and the therapeutic implications of our current knowledge. Resolution of inflammation also requires a functional switch in inflammatory cells biology. Neutrophils are classically considered to be essential players in the host defense against invading pathogens. However, several investigations have shown that impairment of neutrophil migration to the site of infection, also referred to as neutrophil paralysis, occurs during severe sepsis, resulting in an inability of the host to contain and eliminate the infection. On the other hand, the neutrophil antibacterial arsenal contributes to tissue damage and the development of organ dysfunction during sepsis. Sônego et al. provide an overview of the main events in which neutrophils play a beneficial or deleterious role in the outcome of sepsis. Finally, Ferreira et al. provide a comprehensive comparison of the anti-inflammatory effectiveness of two PEGylated TLR7 partial agonists, concerning distinct lung pathological conditions and several routes of administration. The results suggest that the putative clinical application of TMX-302 in lung disorders should be examined with caution because of its direct pro-inflammatory effects. Moreover, in this context, TMX-306 seems to be comparatively more effective and safer than TMX-302, deserving further investigations in drug development particularly for silicosis (Ferreira et al.).

The increasing number of anti-inflammatory drugs best sellers in the pharma market is a clear indication of the relevance of having inflammation under check; nonetheless, there is still a great need for better acting pharmacological tools for the control of inflammation. Indeed, the remarkable success of biological drugs targeting pro-inflammatory cytokines has indicated that tools able to block pro-inflammatory mediators have promising applications. However, there are intrinsic limitations of single targeting drugs because effects frequently vanish, likely due to the well-known redundancy of inflammatory mediators. TIMER has investigated a number of players with potential of developing as innovative targets in this setting and “will not end with the end of dedicated European funding in December 2015, as the legacy of this EU-funded project endures in the preclinical work and in the collaboration that has been fostered,” as his coordinator concluded in the TIMER final meeting.

## Author Contributions

All authors contributed to this editorial.

## Conflict of Interest Statement

The authors declare that the research was conducted in the absence of any commercial or financial relationships that could be construed as a potential conflict of interest.

## Footnotes

^1^http://www.eumbrella.org/.

^2^http://www.iuisonline.org/.

